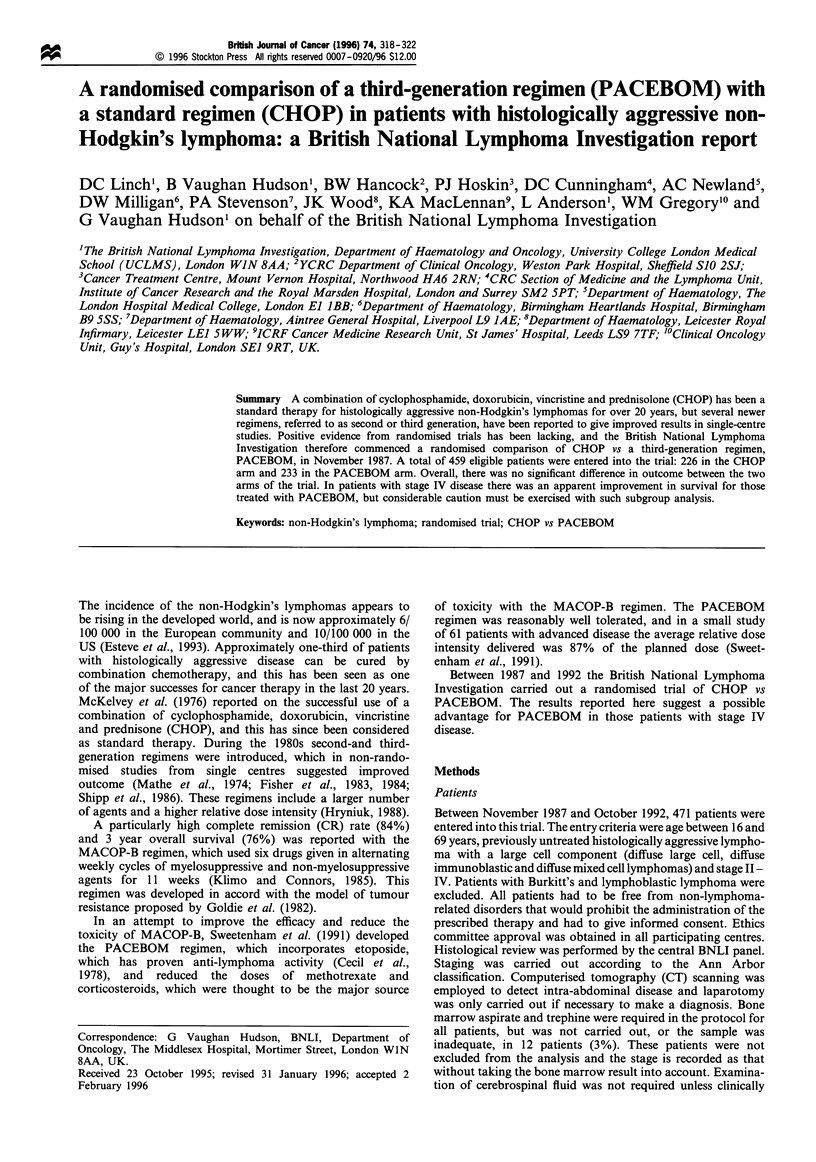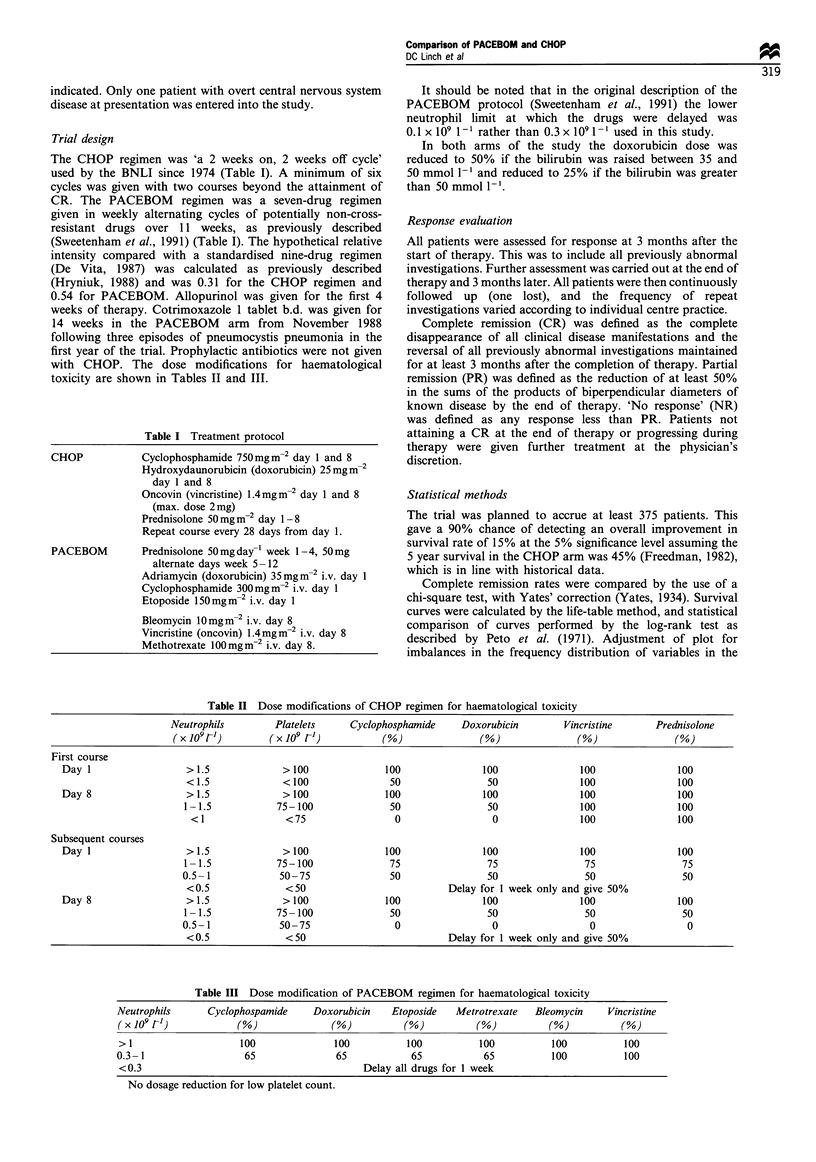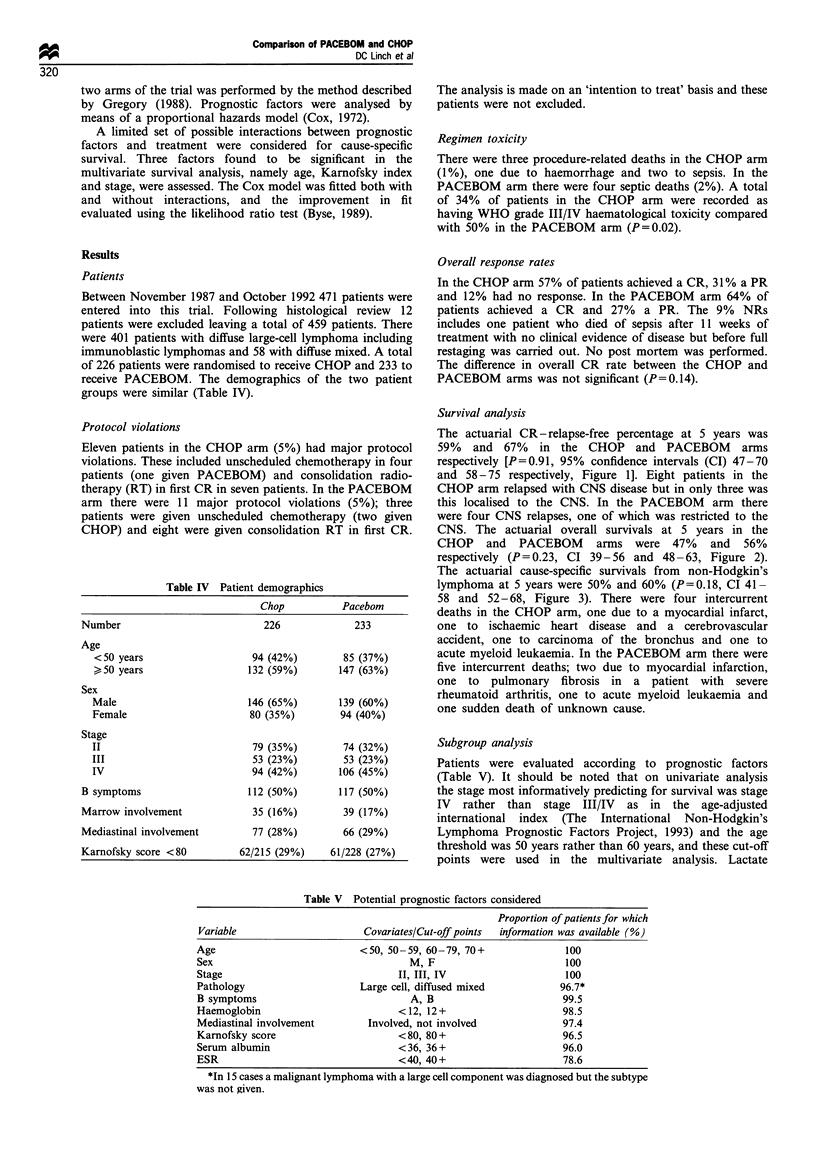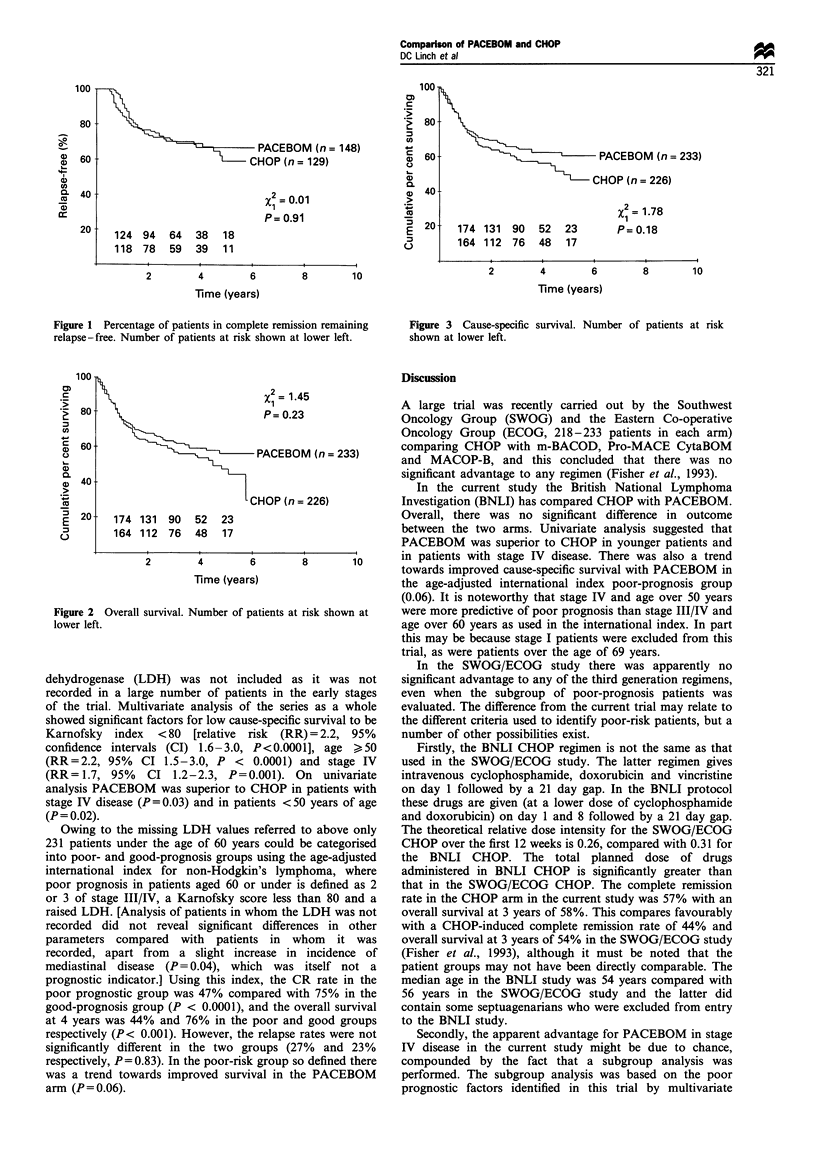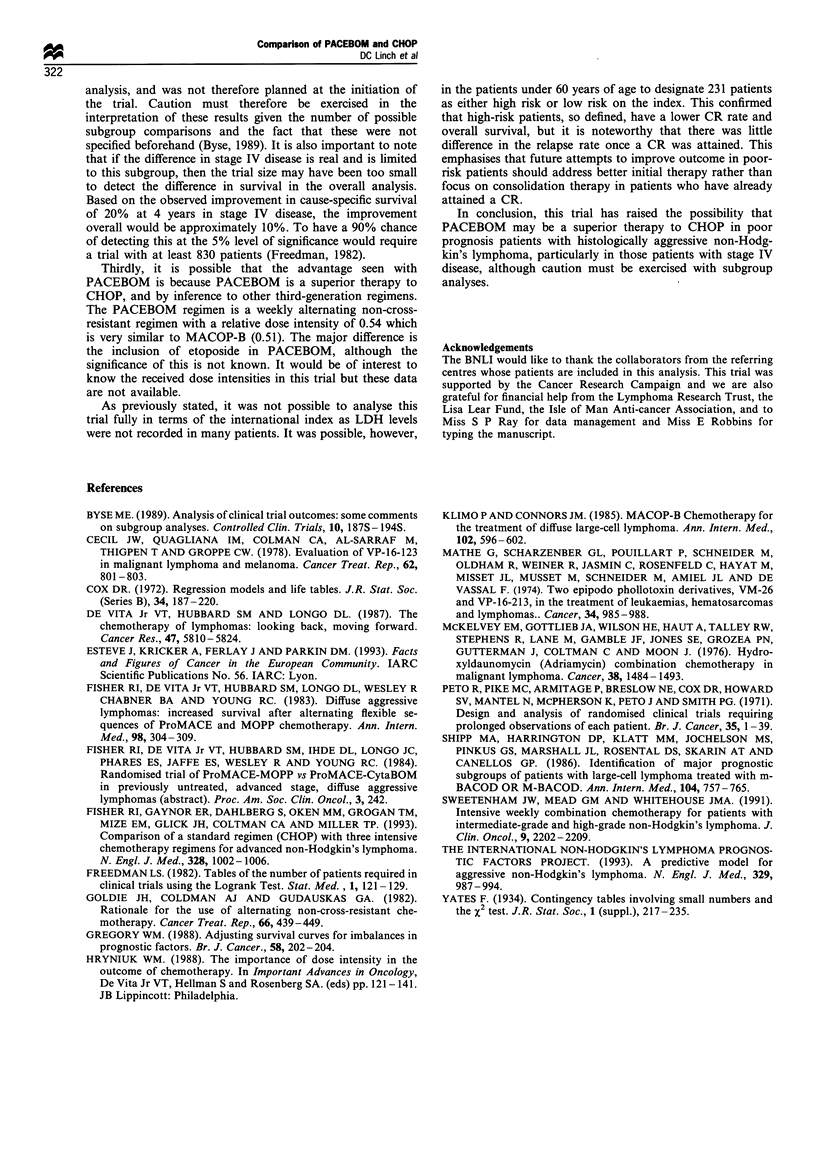# A randomised comparison of a third-generation regimen (PACEBOM) with a standard regimen (CHOP) in patients with histologically aggressive non-Hodgkin's lymphoma: a British National Lymphoma Investigation report.

**DOI:** 10.1038/bjc.1996.360

**Published:** 1996-07

**Authors:** D. C. Linch, B. Vaughan Hudson, B. W. Hancock, P. J. Hoskin, D. C. Cunningham, A. C. Newland, D. W. Milligan, P. A. Stevenson, J. K. Wood, K. A. MacLennan, L. Anderson, W. M. Gregory, G. Vaughan Hudson

**Affiliations:** Department of Haematology and Oncology, University College London Medical School (UCLMS), UK.

## Abstract

A combination of cyclophosphamide, doxorubicin, vincristine and prednisolone (CHOP) has been a standard therapy for histologically aggressive non-Hodgkin's lymphomas for over 20 years, but several newer regimens, referred to as second or third generation, have been reported to give improved results in single-centre studies. Positive evidence from randomised trials has been lacking, and the British National Lymphoma Investigation therefore commenced a randomised comparison of CHOP vs a third-generation regimen, PACEBOM, in November 1987. A total of 459 eligible patients were entered into the trial: 226 in the CHOP arm and 233 in the PACEBOM arm. Overall, there was no significant difference in outcome between the two arms of the trial. In patients with stage IV disease there was an apparent improvement in survival for those treated with PACEBOM, but considerable caution must be exercised with such subgroup analysis.


British Journal of Cancer (1996) 74, 318-322
© 1996 Stockton Press All rights reserved 0007-0920/96 $12.00

### A randomised comparison of a third-generation regimen (PACEBOM) with a standard regimen (CHOP) in patients with histologically aggressive non-Hodgkin's lymphoma: a British National Lymphoma Investigation report

DC Linch[1], B Vaughan Hudson[1], BW Hancock[2], PJ Hoskin[3], DC Cunningham[4], AC Newland[5], DW Milligan[6], PA Stevenson[7], JK Wood[8], KA MacLennan[9], L Anderson[1], WM Gregory[10] and G Vaughan Hudson[1] on behalf of the British National Lymphoma Investigation

[1]*The British National Lymphoma Investigation, Department of Haematology and Oncology, University College London Medical School (UCLMS), London W1N 8AA;* [2]*YCRC Department of Clinical Oncology, Weston Park Hospital, Sheffield S10 2SJ;* [3]*Cancer Treatment Centre, Mount Vernon Hospital, Northwood HA6 2RN;* [4]*CRC Section of Medicine and the Lymphoma Unit, Institute of Cancer Research and the Royal Marsden Hospital, London and Surrey SM2 5PT;* [5]*Department of Haematology, The London Hospital Medical College, London E1 1BB;* [6]*Department of Haematology, Birmingham Heartlands Hospital, Birmingham B9 5SS;* [7]*Department of Haematology, Aintree General Hospital, Liverpool L9 1AE;* [8]*Department of Haematology, Leicester Royal Infirmary, Leicester LE1 5WW;* [9]*ICRF Cancer Medicine Research Unit, St James' Hospital, Leeds LS9 7TF;* [10]*Clinical Oncology Unit, Guy's Hospital, London SE1 9RT, UK.*

**Summary** A combination of cyclophosphamide, doxorubicin, vincristine and prednisolone (CHOP) has been a standard therapy for histologically aggressive non-Hodgkin's lymphomas for over 20 years, but several newer regimens, referred to as second or third generation, have been reported to give improved results in single-centre studies. Positive evidence from randomised trials has been lacking, and the British National Lymphoma Investigation therefore commenced a randomised comparison of CHOP *vs* a third-generation regimen, PACEBOM, in November 1987. A total of 459 eligible patients were entered into the trial: 226 in the CHOP arm and 233 in the PACEBOM arm. Overall, there was no significant difference in outcome between the two arms of the trial. In patients with stage IV disease there was an apparent improvement in survival for those treated with PACEBOM, but considerable caution must be exercised with such subgroup analysis.

**Keywords:** non-Hodgkin's lymphoma; randomised trial; CHOP *vs* PACEBOM

The incidence of the non-Hodgkin's lymphomas appears to be rising in the developed world, and is now approximately 6/100 000 in the European community and 10/100 000 in the US (Esteve *et al.*, 1993). Approximately one-third of patients with histologically aggressive disease can be cured by combination chemotherapy, and this has been seen as one of the major successes for cancer therapy in the last 20 years. McKelvey *et al.* (1976) reported on the successful use of a combination of cyclophosphamide, doxorubicin, vincristine and prednisone (CHOP), and this has since been considered as standard therapy. During the 1980s second-and third-generation regimens were introduced, which in non-randomised studies from single centres suggested improved outcome (Mathe *et al.*, 1974; Fisher *et al.*, 1983, 1984; Shipp *et al.*, 1986). These regimens include a larger number of agents and a higher relative dose intensity (Hryniuk, 1988).

A particularly high complete remission (CR) rate (84%) and 3 year overall survival (76%) was reported with the MACOP-B regimen, which used six drugs given in alternating weekly cycles of myelosuppressive and non-myelosuppressive agents for 11 weeks (Klimo and Connors, 1985). This regimen was developed in accord with the model of tumour resistance proposed by Goldie *et al.* (1982).

In an attempt to improve the efficacy and reduce the toxicity of MACOP-B, Sweetenham *et al.* (1991) developed the PACEBOM regimen, which incorporates etoposide, which has proven anti-lymphoma activity (Cecil *et al.*, 1978), and reduced the doses of methotrexate and corticosteroids, which were thought to be the major source of toxicity with the MACOP-B regimen. The PACEBOM regimen was reasonably well tolerated, and in a small study of 61 patients with advanced disease the average relative dose intensity delivered was 87% of the planned dose (Sweetenham *et al.*, 1991).

Between 1987 and 1992 the British National Lymphoma Investigation carried out a randomised trial of CHOP *vs* PACEBOM. The results reported here suggest a possible advantage for PACEBOM in those patients with stage IV disease.

### Methods

### *Patients*

Between November 1987 and October 1992, 471 patients were entered into this trial. The entry criteria were age between 16 and 69 years, previously untreated histologically aggressive lymphoma with a large cell component (diffuse large cell, diffuse immunoblastic and diffuse mixed cell lymphomas) and stage II–IV. Patients with Burkitt's and lymphoblastic lymphoma were excluded. All patients had to be free from non-lymphoma-related disorders that would prohibit the administration of the prescribed therapy and had to give informed consent. Ethics committee approval was obtained in all participating centres. Histological review was performed by the central BNLI panel. Staging was carried out according to the Ann Arbor classification. Computerised tomography (CT) scanning was employed to detect intra-abdominal disease and laparotomy was only carried out if necessary to make a diagnosis. Bone marrow aspirate and trephine were required in the protocol for all patients, but was not carried out, or the sample was inadequate, in 12 patients (3%). These patients were not excluded from the analysis and the stage is recorded as that without taking the bone marrow result into account. Examination of cerebrospinal fluid was not required unless clinically

Correspondence: G Vaughan Hudson, BNLI, Department of Oncology, The Middlesex Hospital, Mortimer Street, London W1N 8AA, UK.
Received 23 October 1995; revised 31 January 1996; accepted 2 February 1996

indicated. Only one patient with overt central nervous system disease at presentation was entered into the study.

*Trial design*

The CHOP regimen was 'a 2 weeks on, 2 weeks off cycle' used by the BNLI since 1974 (Table I). A minimum of six cycles was given with two courses beyond the attainment of CR. The PACEBOM regimen was a seven-drug regimen given in weekly alternating cycles of potentially non-cross-resistant drugs over 11 weeks, as previously described (Sweetenham *et al.*, 1991) (Table I). The hypothetical relative intensity compared with a standardised nine-drug regimen (De Vita, 1987) was calculated as previously described (Hryniuk, 1988) and was 0.31 for the CHOP regimen and 0.54 for PACEBOM. Allopurinol was given for the first 4 weeks of therapy. Cotrimoxazole 1 tablet b.d. was given for 14 weeks in the PACEBOM arm from November 1988 following three episodes of pneumocystis pneumonia in the first year of the trial. Prophylactic antibiotics were not given with CHOP. The dose modifications for haematological toxicity are shown in Tables II and III.

**Table I** Treatment protocol

| | |
|---|---|
| CHOP | Cyclophosphamide 750 mg m$^{-2}$ day 1 and 8 |
| | Hydroxydaunorubicin (doxorubicin) 25 mg m$^{-2}$ day 1 and 8 |
| | Oncovin (vincristine) 1.4 mg m$^{-2}$ day 1 and 8 (max. dose 2 mg) |
| | Prednisolone 50 mg m$^{-2}$ day 1–8 |
| | Repeat course every 28 days from day 1. |
| PACEBOM | Prednisolone 50 mg day$^{-1}$ week 1–4, 50 mg alternate days week 5–12 |
| | Adriamycin (doxorubicin) 35 mg m$^{-2}$ i.v. day 1 |
| | Cyclophosphamide 300 mg m$^{-2}$ i.v. day 1 |
| | Etoposide 150 mg m$^{-2}$ i.v. day 1 |
| | Bleomycin 10 mg m$^{-2}$ i.v. day 8 |
| | Vincristine (oncovin) 1.4 mg m$^{-2}$ i.v. day 8 |
| | Methotrexate 100 mg m$^{-2}$ i.v. day 8. |

It should be noted that in the original description of the PACEBOM protocol (Sweetenham *et al.*, 1991) the lower neutrophil limit at which the drugs were delayed was $0.1 \times 10^9 \, l^{-1}$ rather than $0.3 \times 10^9 \, l^{-1}$ used in this study.

In both arms of the study the doxorubicin dose was reduced to 50% if the bilirubin was raised between 35 and 50 mmol $l^{-1}$ and reduced to 25% if the bilirubin was greater than 50 mmol $l^{-1}$.

*Response evaluation*

All patients were assessed for response at 3 months after the start of therapy. This was to include all previously abnormal investigations. Further assessment was carried out at the end of therapy and 3 months later. All patients were then continuously followed up (one lost), and the frequency of repeat investigations varied according to individual centre practice.

Complete remission (CR) was defined as the complete disappearance of all clinical disease manifestations and the reversal of all previously abnormal investigations maintained for at least 3 months after the completion of therapy. Partial remission (PR) was defined as the reduction of at least 50% in the sums of the products of biperpendicular diameters of known disease by the end of therapy. 'No response' (NR) was defined as any response less than PR. Patients not attaining a CR at the end of therapy or progressing during therapy were given further treatment at the physician's discretion.

*Statistical methods*

The trial was planned to accrue at least 375 patients. This gave a 90% chance of detecting an overall improvement in survival rate of 15% at the 5% significance level assuming the 5 year survival in the CHOP arm was 45% (Freedman, 1982), which is in line with historical data.

Complete remission rates were compared by the use of a chi-square test, with Yates' correction (Yates, 1934). Survival curves were calculated by the life-table method, and statistical comparison of curves performed by the log-rank test as described by Peto *et al.* (1971). Adjustment of plot for imbalances in the frequency distribution of variables in the

**Table II** Dose modifications of CHOP regimen for haematological toxicity

| | *Neutrophils* ($\times 10^9 \, l^{-1}$) | *Platelets* ($\times 10^9 \, l^{-1}$) | *Cyclophosphamide* (%) | *Doxorubicin* (%) | *Vincristine* (%) | *Prednisolone* (%) |
|---|---|---|---|---|---|---|
| First course | | | | | | |
| Day 1 | >1.5 | >100 | 100 | 100 | 100 | 100 |
| | <1.5 | <100 | 50 | 50 | 100 | 100 |
| Day 8 | >1.5 | >100 | 100 | 100 | 100 | 100 |
| | 1–1.5 | 75–100 | 50 | 50 | 100 | 100 |
| | <1 | <75 | 0 | 0 | 100 | 100 |
| Subsequent courses | | | | | | |
| Day 1 | >1.5 | >100 | 100 | 100 | 100 | 100 |
| | 1–1.5 | 75–100 | 75 | 75 | 75 | 75 |
| | 0.5–1 | 50–75 | 50 | 50 | 50 | 50 |
| | <0.5 | <50 | | Delay for 1 week only and give 50% | | |
| Day 8 | >1.5 | >100 | 100 | 100 | 100 | 100 |
| | 1–1.5 | 75–100 | 50 | 50 | 50 | 50 |
| | 0.5–1 | 50–75 | 0 | 0 | 0 | 0 |
| | <0.5 | <50 | | Delay for 1 week only and give 50% | | |

**Table III** Dose modification of PACEBOM regimen for haematological toxicity

| *Neutrophils* ($\times 10^9 \, l^{-1}$) | *Cyclophospamide* (%) | *Doxorubicin* (%) | *Etoposide* (%) | *Metrotrexate* (%) | *Bleomycin* (%) | *Vincristine* (%) |
|---|---|---|---|---|---|---|
| >1 | 100 | 100 | 100 | 100 | 100 | 100 |
| 0.3–1 | 65 | 65 | 65 | 65 | 100 | 100 |
| <0.3 | | | Delay all drugs for 1 week | | | |

No dosage reduction for low platelet count.

two arms of the trial was performed by the method described by Gregory (1988). Prognostic factors were analysed by means of a proportional hazards model (Cox, 1972).

A limited set of possible interactions between prognostic factors and treatment were considered for cause-specific survival. Three factors found to be significant in the multivariate survival analysis, namely age, Karnofsky index and stage, were assessed. The Cox model was fitted both with and without interactions, and the improvement in fit evaluated using the likelihood ratio test (Byse, 1989).

### Results

### Patients

Between November 1987 and October 1992 471 patients were entered into this trial. Following histological review 12 patients were excluded leaving a total of 459 patients. There were 401 patients with diffuse large-cell lymphoma including immunoblastic lymphomas and 58 with diffuse mixed. A total of 226 patients were randomised to receive CHOP and 233 to receive PACEBOM. The demographics of the two patient groups were similar (Table IV).

### Protocol violations

Eleven patients in the CHOP arm (5%) had major protocol violations. These included unscheduled chemotherapy in four patients (one given PACEBOM) and consolidation radiotherapy (RT) in first CR in seven patients. In the PACEBOM arm there were 11 major protocol violations (5%); three patients were given unscheduled chemotherapy (two given CHOP) and eight were given consolidation RT in first CR.

**Table IV** Patient demographics

| | *Chop* | *Pacebom* |
|---|---|---|
| Number | 226 | 233 |
| Age | | |
| <50 years | 94 (42%) | 85 (37%) |
| ⩾50 years | 132 (59%) | 147 (63%) |
| Sex | | |
| Male | 146 (65%) | 139 (60%) |
| Female | 80 (35%) | 94 (40%) |
| Stage | | |
| II | 79 (35%) | 74 (32%) |
| III | 53 (23%) | 53 (23%) |
| IV | 94 (42%) | 106 (45%) |
| B symptoms | 112 (50%) | 117 (50%) |
| Marrow involvement | 35 (16%) | 39 (17%) |
| Mediastinal involvement | 77 (28%) | 66 (29%) |
| Karnofsky score <80 | 62/215 (29%) | 61/228 (27%) |

The analysis is made on an 'intention to treat' basis and these patients were not excluded.

### Regimen toxicity

There were three procedure-related deaths in the CHOP arm (1%), one due to haemorrhage and two to sepsis. In the PACEBOM arm there were four septic deaths (2%). A total of 34% of patients in the CHOP arm were recorded as having WHO grade III/IV haematological toxicity compared with 50% in the PACEBOM arm ($P=0.02$).

### Overall response rates

In the CHOP arm 57% of patients achieved a CR, 31% a PR and 12% had no response. In the PACEBOM arm 64% of patients achieved a CR and 27% a PR. The 9% NRs includes one patient who died of sepsis after 11 weeks of treatment with no clinical evidence of disease but before full restaging was carried out. No post mortem was performed. The difference in overall CR rate between the CHOP and PACEBOM arms was not significant ($P=0.14$).

### Survival analysis

The actuarial CR–relapse-free percentage at 5 years was 59% and 67% in the CHOP and PACEBOM arms respectively [$P=0.91$, 95% confidence intervals (CI) 47–70 and 58–75 respectively, Figure 1]. Eight patients in the CHOP arm relapsed with CNS disease but in only three was this localised to the CNS. In the PACEBOM arm there were four CNS relapses, one of which was restricted to the CNS. The actuarial overall survivals at 5 years in the CHOP and PACEBOM arms were 47% and 56% respectively ($P=0.23$, CI 39–56 and 48–63, Figure 2). The actuarial cause-specific survivals from non-Hodgkin's lymphoma at 5 years were 50% and 60% ($P=0.18$, CI 41–58 and 52–68, Figure 3). There were four intercurrent deaths in the CHOP arm, one due to a myocardial infarct, one to ischaemic heart disease and a cerebrovascular accident, one to carcinoma of the bronchus and one to acute myeloid leukaemia. In the PACEBOM arm there were five intercurrent deaths; two due to myocardial infarction, one to pulmonary fibrosis in a patient with severe rheumatoid arthritis, one to acute myeloid leukaemia and one sudden death of unknown cause.

### Subgroup analysis

Patients were evaluated according to prognostic factors (Table V). It should be noted that on univariate analysis the stage most informatively predicting for survival was stage IV rather than stage III/IV as in the age-adjusted international index (The International Non-Hodgkin's Lymphoma Prognostic Factors Project, 1993) and the age threshold was 50 years rather than 60 years, and these cut-off points were used in the multivariate analysis. Lactate

**Table V** Potential prognostic factors considered

| *Variable* | *Covariates/Cut-off points* | *Proportion of patients for which information was available (%)* |
|---|---|---|
| Age | <50, 50–59, 60–79, 70+ | 100 |
| Sex | M, F | 100 |
| Stage | II, III, IV | 100 |
| Pathology | Large cell, diffused mixed | 96.7* |
| B symptoms | A, B | 99.5 |
| Haemoglobin | <12, 12+ | 98.5 |
| Mediastinal involvement | Involved, not involved | 97.4 |
| Karnofsky score | <80, 80+ | 96.5 |
| Serum albumin | <36, 36+ | 96.0 |
| ESR | <40, 40+ | 78.6 |

*In 15 cases a malignant lymphoma with a large cell component was diagnosed but the subtype was not given.

**Figure 1** Percentage of patients in complete remission remaining relapse-free. Number of patients at risk shown at lower left.

**Figure 2** Overall survival. Number of patients at risk shown at lower left.

dehydrogenase (LDH) was not included as it was not recorded in a large number of patients in the early stages of the trial. Multivariate analysis of the series as a whole showed significant factors for low cause-specific survival to be Karnofsky index <80 [relative risk (RR) = 2.2, 95% confidence intervals (CI) 1.6–3.0, $P$<0.0001], age ⩾50 (RR = 2.2, 95% CI 1.5–3.0, $P$ < 0.0001) and stage IV (RR = 1.7, 95% CI 1.2–2.3, $P$ = 0.001). On univariate analysis PACEBOM was superior to CHOP in patients with stage IV disease ($P$ = 0.03) and in patients <50 years of age ($P$ = 0.02).

Owing to the missing LDH values referred to above only 231 patients under the age of 60 years could be categorised into poor- and good-prognosis groups using the age-adjusted international index for non-Hodgkin's lymphoma, where poor prognosis in patients aged 60 or under is defined as 2 or 3 of stage III/IV, a Karnofsky score less than 80 and a raised LDH. [Analysis of patients in whom the LDH was not recorded did not reveal significant differences in other parameters compared with patients in whom it was recorded, apart from a slight increase in incidence of mediastinal disease ($P$ = 0.04), which was itself not a prognostic indicator.] Using this index, the CR rate in the poor prognostic group was 47% compared with 75% in the good-prognosis group ($P$ < 0.0001), and the overall survival at 4 years was 44% and 76% in the poor and good groups respectively ($P$< 0.001). However, the relapse rates were not significantly different in the two groups (27% and 23% respectively, $P$ = 0.83). In the poor-risk group so defined there was a trend towards improved survival in the PACEBOM arm ($P$ = 0.06).

**Figure 3** Cause-specific survival. Number of patients at risk shown at lower left.

### Discussion

A large trial was recently carried out by the Southwest Oncology Group (SWOG) and the Eastern Co-operative Oncology Group (ECOG, 218–233 patients in each arm) comparing CHOP with m-BACOD, Pro-MACE CytaBOM and MACOP-B, and this concluded that there was no significant advantage to any regimen (Fisher *et al.*, 1993).

In the current study the British National Lymphoma Investigation (BNLI) has compared CHOP with PACEBOM. Overall, there was no significant difference in outcome between the two arms. Univariate analysis suggested that PACEBOM was superior to CHOP in younger patients and in patients with stage IV disease. There was also a trend towards improved cause-specific survival with PACEBOM in the age-adjusted international index poor-prognosis group (0.06). It is noteworthy that stage IV and age over 50 years were more predictive of poor prognosis than stage III/IV and age over 60 years as used in the international index. In part this may be because stage I patients were excluded from this trial, as were patients over the age of 69 years.

In the SWOG/ECOG study there was apparently no significant advantage to any of the third generation regimens, even when the subgroup of poor-prognosis patients was evaluated. The difference from the current trial may relate to the different criteria used to identify poor-risk patients, but a number of other possibilities exist.

Firstly, the BNLI CHOP regimen is not the same as that used in the SWOG/ECOG study. The latter regimen gives intravenous cyclophosphamide, doxorubicin and vincristine on day 1 followed by a 21 day gap. In the BNLI protocol these drugs are given (at a lower dose of cyclophosphamide and doxorubicin) on day 1 and 8 followed by a 21 day gap. The theoretical relative dose intensity for the SWOG/ECOG CHOP over the first 12 weeks is 0.26, compared with 0.31 for the BNLI CHOP. The total planned dose of drugs administered in BNLI CHOP is significantly greater than that in the SWOG/ECOG CHOP. The complete remission rate in the CHOP arm in the current study was 57% with an overall survival at 3 years of 58%. This compares favourably with a CHOP-induced complete remission rate of 44% and overall survival at 3 years of 54% in the SWOG/ECOG study (Fisher *et al.*, 1993), although it must be noted that the patient groups may not have been directly comparable. The median age in the BNLI study was 54 years compared with 56 years in the SWOG/ECOG study and the latter did contain some septuagenarians who were excluded from entry to the BNLI study.

Secondly, the apparent advantage for PACEBOM in stage IV disease in the current study might be due to chance, compounded by the fact that a subgroup analysis was performed. The subgroup analysis was based on the poor prognostic factors identified in this trial by multivariate analysis, and was not therefore planned at the initiation of the trial. Caution must therefore be exercised in the interpretation of these results given the number of possible subgroup comparisons and the fact that these were not specified beforehand (Byse, 1989). It is also important to note that if the difference in stage IV disease is real and is limited to this subgroup, then the trial size may have been too small to detect the difference in survival in the overall analysis. Based on the observed improvement in cause-specific survival of 20% at 4 years in stage IV disease, the improvement overall would be approximately 10%. To have a 90% chance of detecting this at the 5% level of significance would require a trial with at least 830 patients (Freedman, 1982).

Thirdly, it is possible that the advantage seen with PACEBOM is because PACEBOM is a superior therapy to CHOP, and by inference to other third-generation regimens. The PACEBOM regimen is a weekly alternating non-cross-resistant regimen with a relative dose intensity of 0.54 which is very similar to MACOP-B (0.51). The major difference is the inclusion of etoposide in PACEBOM, although the significance of this is not known. It would be of interest to know the received dose intensities in this trial but these data are not available.

As previously stated, it was not possible to analyse this trial fully in terms of the international index as LDH levels were not recorded in many patients. It was possible, however, in the patients under 60 years of age to designate 231 patients as either high risk or low risk on the index. This confirmed that high-risk patients, so defined, have a lower CR rate and overall survival, but it is noteworthy that there was little difference in the relapse rate once a CR was attained. This emphasises that future attempts to improve outcome in poor-risk patients should address better initial therapy rather than focus on consolidation therapy in patients who have already attained a CR.

In conclusion, this trial has raised the possibility that PACEBOM may be a superior therapy to CHOP in poor prognosis patients with histologically aggressive non-Hodgkin's lymphoma, particularly in those patients with stage IV disease, although caution must be exercised with subgroup analyses.

**Acknowledgements**

The BNLI would like to thank the collaborators from the referring centres whose patients are included in this analysis. This trial was supported by the Cancer Research Campaign and we are also grateful for financial help from the Lymphoma Research Trust, the Lisa Lear Fund, the Isle of Man Anti-cancer Association, and to Miss S P Ray for data management and Miss E Robbins for typing the manuscript.

### References

BYSE ME. (1989). Analysis of clinical trial outcomes: some comments on subgroup analyses. *Controlled Clin. Trials*, **10**, 187S-194S.

CECIL JW, QUAGLIANA IM, COLMAN CA, AL-SARRAF M, THIGPEN T AND GROPPE CW. (1978). Evaluation of VP-16-123 in malignant lymphoma and melanoma. *Cancer Treat. Rep.*, **62**, 801-803.

COX DR. (1972). Regression models and life tables. *J.R. Stat. Soc.* (Series B), **34**, 187-220.

DE VITA Jr VT, HUBBARD SM AND LONGO DL. (1987). The chemotherapy of lymphomas: looking back, moving forward. *Cancer Res.*, **47**, 5810-5824.

ESTEVE J, KRICKER A, FERLAY J AND PARKIN DM. (1993). *Facts and Figures of Cancer in the European Community*. IARC Scientific Publications No. 56. IARC: Lyon.

FISHER RI, DE VITA Jr VT, HUBBARD SM, LONGO DL, WESLEY R CHABNER BA AND YOUNG RC. (1983). Diffuse aggressive lymphomas: increased survival after alternating flexible sequences of ProMACE and MOPP chemotherapy. *Ann. Intern. Med.*, **98**, 304-309.

FISHER RI, DE VITA Jr VT, HUBBARD SM, IHDE DL, LONGO JC, PHARES ES, JAFFE ES, WESLEY R AND YOUNG RC. (1984). Randomised trial of ProMACE-MOPP *vs* ProMACE-CytaBOM in previously untreated, advanced stage, diffuse aggressive lymphomas (abstract). *Proc. Am. Soc. Clin. Oncol.*, **3**, 242.

FISHER RI, GAYNOR ER, DAHLBERG S, OKEN MM, GROGAN TM, MIZE EM, GLICK JH, COLTMAN CA AND MILLER TP. (1993). Comparison of a standard regimen (CHOP) with three intensive chemotherapy regimens for advanced non-Hodgkin's lymphoma. *N. Engl. J. Med.*, **328**, 1002-1006.

FREEDMAN LS. (1982). Tables of the number of patients required in clinical trials using the Logrank Test. *Stat. Med.* , **1**, 121-129.

GOLDIE JH, COLDMAN AJ AND GUDAUSKAS GA. (1982). Rationale for the use of alternating non-cross-resistant chemotherapy. *Cancer Treat. Rep.*, **66**, 439-449.

GREGORY WM. (1988). Adjusting survival curves for imbalances in prognostic factors. *Br. J. Cancer.*, **58**, 202-204.

HRYNIUK WM. (1988). The importance of dose intensity in the outcome of chemotherapy. In *Important Advances in Oncology*, De Vita Jr VT, Hellman S and Rosenberg SA. (eds) pp. 121-141. JB Lippincott: Philadelphia.

KLIMO P AND CONNORS JM. (1985). MACOP-B Chemotherapy for the treatment of diffuse large-cell lymphoma. *Ann. Intern. Med.*, **102**, 596-602.

MATHE G, SCHARZENBER GL, POUILLART P, SCHNEIDER M, OLDHAM R, WEINER R, JASMIN C, ROSENFELD C, HAYAT M, MISSET JL, MUSSET M, SCHNEIDER M, AMIEL JL AND DE VASSAL F. (1974). Two epipodo phollotoxin derivatives, VM-26 and VP-16-213, in the treatment of leukaemias, hematosarcomas and lymphomas.. *Cancer*, **34**, 985-988.

MCKELVEY EM, GOTTLIEB JA, WILSON HE, HAUT A, TALLEY RW, STEPHENS R, LANE M, GAMBLE JF, JONES SE, GROZEA PN, GUTTERMAN J, COLTMAN C AND MOON J. (1976). Hydroxyldaunomycin (Adriamycin) combination chemotherapy in malignant lymphoma. *Cancer*, **38**, 1484-1493.

PETO R, PIKE MC, ARMITAGE P, BRESLOW NE, COX DR, HOWARD SV, MANTEL N, MCPHERSON K, PETO J AND SMITH PG. (1971). Design and analysis of randomised clinical trials requiring prolonged observations of each patient. *Br. J. Cancer*, **35**, 1-39.

SHIPP MA, HARRINGTON DP, KLATT MM, JOCHELSON MS, PINKUS GS, MARSHALL JL, ROSENTAL DS, SKARIN AT AND CANELLOS GP. (1986). Identification of major prognostic subgroups of patients with large-cell lymphoma treated with m-BACOD OR M-BACOD. *Ann. Intern. Med.*, **104**, 757-765.

SWEETENHAM JW, MEAD GM AND WHITEHOUSE JMA. (1991). Intensive weekly combination chemotherapy for patients with intermediate-grade and high-grade non-Hodgkin's lymphoma. *J. Clin. Oncol.*, **9**, 2202-2209.

THE INTERNATIONAL NON-HODGKIN'S LYMPHOMA PROGNOSTIC FACTORS PROJECT. (1993). A predictive model for aggressive non-Hodgkin's lymphoma. *N. Engl. J. Med.*, **329**, 987-994.

YATES F. (1934). Contingency tables involving small numbers and the $\chi^2$ test. *J.R. Stat. Soc.*, **1** (suppl.), 217-235.